# Epidemiology of non‐alcoholic fatty liver disease and non‐alcoholic steatohepatitis in Japan: A focused literature review

**DOI:** 10.1002/jgh3.12349

**Published:** 2020-05-05

**Authors:** Yuichiro Eguchi, Gabriel Wong, Emma (I‐Heng) Lee, Omar Akhtar, Ricardo Lopes, Yoshio Sumida

**Affiliations:** ^1^ Liver Center, Saga University Hospital, Saga University Saga Japan; ^2^ Gilead Sciences, Inc. Foster City California USA; ^3^ Amaris London UK; ^4^ Amaris Barcelona Spain; ^5^ Division of Hepatology and Pancreatology, Department of Internal Medicine Aichi Medical University Nagakute Japan

**Keywords:** cirrhosis, epidemiology, morbidity, non‐alcoholic fatty liver disease, non‐alcoholic steatohepatitis

## Abstract

Non‐alcoholic fatty liver disease (NAFLD) and non‐alcoholic steatohepatitis (NASH) represent a growing unmet medical need and an increasingly prevalent cause of cirrhosis, hepatocellular carcinoma (HCC), and death in Japan. The aim of this review was to characterize the epidemiology of NAFLD and NASH in Japan. An English and Japanese literature search was conducted in PubMed, Embase, and ICHUSHI Web, identifying 6553 studies, 67 of which were included. Prevalence of NAFLD in the Japanese population rose from the early 1990s (12.6–12.9%) to the early 2000s (24.6–34.7% of the population). Japanese NASH prevalence is estimated to be 1.9–2.7%. NAFLD and NASH are more common among males than females; however, females experience more severe disease than males. While obese patients had higher prevalence of NAFLD/NASH, nonobese individuals (body mass index [BMI] <25 kg/m^2^) consistently comprised 20% to >35% of NAFLD and NASH patients. The evidence shows that, despite obesity being linked with worse disease stages, “lean‐NASH” also plays an important role in NASH epidemiology. Besides obesity, diabetes and metabolic syndrome appeared to be reliably associated with disease severity. The prevalence of advanced fibrosis or cirrhotic disease was the highest in patients with NASH‐HCC (44–80% with stage F3/F4 disease), while 21–50% of patients with NASH had F3/F4 disease. NAFLD/NASH is common in the Japanese population, and the prevalence of these conditions has tripled in the last two decades. Furthermore, these NAFLD/NASH patients have a high comorbidity burden. Early and efficient identification of safe and effective treatments for NAFLD/NASH patients is urgently needed.

## Graphical abstract

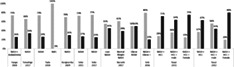

This review characterizes the epidemiology and morbidity of NAFLD and NASH in Japan. The prevalence of both conditions has been increasing in the past three decades and the disease progression is associated with the presence of obesity, diabetes mellitus and metabolic syndrome. The prevalence of advanced fibrosis or cirrhotic disease was highest in patients with NASH‐HCC compared with regular NASH patients: 44%‐80% versus 21%–50%.

## Introduction

Non‐alcoholic fatty liver disease (NAFLD) is becoming one of the most common forms of liver disease worldwide, with a global prevalence rate of approximately 25%.^1^

Recent evidence on genetic polymorphisms suggests that Asian populations are predisposed to NAFLD and its most progressive form, non‐alcoholic steatohepatitis (NASH).^2^ However, data also show that it is unlikely that Asian patients have less severe outcomes compared with other populations.^2^ It is estimated that 10% of NASH patients will develop decompensated liver disease over 13 years, and 10–25% will develop cirrhosis over 5–9 years.^2^ For cirrhotic patients, the risk of developing portal hypertension is 17, 23, and 52% at 1, 3, and 10 years, respectively. Patients with NASH‐related cirrhosis have an increased risk of developing hepatocellular carcinoma (HCC).^3^ The annual incidence of HCC in these patients is estimated to range from 2.8 to 12.8%.^3^

NAFLD and NASH are often associated with metabolic disorders such as type 2 diabetes mellitus (T2DM), hypertension, dyslipidemia, hyperlipidemia, metabolic syndrome, and—more commonly—obesity.^4^ Despite obesity being highly prevalent among NAFLD/NASH patients, there is still a considerable proportion of nonobese NAFLD/NASH patients.^4^ This condition is often referred to as lean‐NASH, also denoted as nonobese NASH (body mass index [BMI] <25 kg/m^2^ in Japan) or nonoverweight NASH (BMI <22 kg/m^2^ in Japan).^4^

Negative changes in diet and lifestyle have led to a dramatic increase in the prevalence of diabetes mellitus (DM), obesity, and metabolic syndrome in Western countries and many Asian countries, which has led to a significant increase in the incidence and prevalence of NAFLD.^5^ The prevalence of NAFLD is estimated to be 12–30% in Asian countries, which is consistent with estimates for Europe and North America: 20–40%.^5^, ^6^, ^7^ In Japan, NAFLD prevalence is estimated to range from 29.7 to 35.1%, making NAFLD an important condition to consider from a public health perspective.^8^, ^9^

NAFLD and NASH are associated with a considerable economic burden, mostly due to the high disease burden related to T2DM, obesity, and cirrhosis.^10^ A modeling study published in 2016 estimated the annual burden associated with all incident and prevalent NAFLD cases in the United States, Germany, France, Italy, and the United Kingdom.^10^ According to the authors, direct and indirect costs were estimated at US$103 billion in the United States ($1613 per patient) and at €35 billion in the four Europe countries (from €354 to €1163 per patient). NASH‐related advanced fibrosis (F3/4) accounted for more than half of the total costs.^10^

Given the relevant epidemiologic and economic burden of NAFLD/NASH, it is of interest to characterize the epidemiology and natural history of these conditions, with particular focus on F3/4 stages of the disease. The aim of this structured review is to characterize the epidemiology and natural history of NAFLD and NASH in Japan by answering the following research questions:

- What are the incidence and prevalence data available for the NAFLD/NASH population in Japan, especially for F3 and F4 disease?
- What are the comorbidities observed among NASH patients, particularly in F3 and F4 stages?
- How do comorbid obesity, T2DM, cardiovascular disease, and renal disease alter disease progression?

## Methods

To identify relevant studies, electronic searches of both English and Japanese literature were conducted in PubMed, Embase, and Ichushi Web, in addition to hand searches of relevant conferences and clinical and diagnostic guidelines (PICOS framework is available in Appendix S1). Keywords and subject headings reflecting NAFLD and NASH and the Japanese population were used in combination to identify relevant studies.

Studies were defined as eligible for inclusion if the following criteria were met: (i) Language of interest: English or Japanese; (ii) Type of study: observational studies; (iii) Study population: Japanese patients with NAFLD or NASH; (iv) Outcomes of interest: incidence of NAFLD and NASH, prevalence of NAFLD and NASH, and prevalence of comorbidities (obesity, DM, hypertension, dyslipidemia, and metabolic syndrome) among NAFLD and NASH patients and distribution of mild (F0/2) and advanced fibrosis (F3/4); And (v) Publication date: 2005–2018 (date of search). Studies were excluded if they failed to meet the above criteria.

Study selection was initially performed by review of titles and abstracts of identified studies. Full‐text review was performed on potentially relevant studies to determine final inclusion in the literature review. Information from the final set of studies regarding study and patient characteristics and the outcomes reported were extracted into standardized forms.

## Results

Using the search criteria presented in Appendix S1, 6553 studies were identified. After title and abstract screening according to predefined criteria, 248 studies were considered eligible for full‐text review, and based on the Population Intervention Comparison Outcomes Study type (PICOS) framework, 67 of these studies were included in this review (Appendix S1). The Preferred Reporting Items for Systematic Reviews and Meta‐Analyses (PRISMA) diagram summarizing the inclusion/exclusion process is displayed in Appendix S2.

Across the 67 studies included in this review, 52.2% (*n* = 35) were retrospective cohort studies, 26.9% (*n* = 18) were prospective cohort studies, and 20.9% (*n* = 14) were cross‐sectional cohort studies.^6^, ^7^, ^8^, ^9^, ^11^, ^12^, ^13^, ^14^, ^15^, ^16^, ^17^, ^18^, ^19^, ^20^, ^21^, ^22^, ^23^, ^24^, ^25^, ^26^, ^27^, ^28^, ^29^, ^30^, ^31^, ^32^, ^33^, ^34^, ^35^, ^36^, ^37^, ^38^, ^39^, ^40^, ^41^, ^42^, ^43^, ^44^, ^45^, ^46^, ^47^, ^48^, ^49^, ^50^, ^51^, ^52^, ^53^, ^54^, ^55^, ^56^, ^57^, ^58^, ^59^, ^60^, ^61^, ^62^, ^63^, ^64^, ^65^, ^66^, ^67^, ^68^, ^69^ Details on data source, geographic location, study period, population, and sample size are displayed in Appendix S3.

### *Prevalence of* *NAFLD/NASH*

The prevalence of NAFLD in Japan was reported in seven studies. Evidence shows that the prevalence of NAFLD has increased at least twofold since the early 1990s, with current estimates ranging from 24.6 to 34.7% of the general Japanese population compared to 12.6–12.9% in the early 1990s, suggesting that NAFLD is highly prevalent.^6^, ^8^ A brief summary of the studies reporting the prevalence of NAFLD is presented in Table 1.

**Table 1:** Prevalence of NAFLD in Japan

Study	Region	Sample size	Mean age	Dates of study	Method of diagnosis	NAFLD prevalence
Eguchi *et al*.^8^	Saga, Hiroshima and Kochi prefectures	5075	50.0	2009–2010	Abdominal US	29.7%
		M: 51.76%				M: 41%
		F: 48.24%				F: 17.7%
Hamaguchi *et al*.^11^	Gifu prefecture	4401	47.6	2001–2003	Abdominal US	18%
		M: 58.44%				M: 24.65%
		F: 41.56%				F: 17.7%
Hamaguchi *et al*.^47^	Gifu prefecture	1647	47.8	1998	Abdominal US	19%
		M: 59.50%				
		F: 40.50%				
Jimba *et al*.^7^	Saitama prefecture	1950	49	2002–2003	Abdominal US	29%
		M: 69%				M: 40%
		F: 31%				F: 22%
Komeda *et al*.^6^	Kyoto prefecture	NR (>1000 patients/year)	NR	1995–2004	Abdominal US	1995: 12.9%
						2004: 34.7%
Nishioji *et al*.^9^	Kyoto prefecture	3271	56.9	2011–2012	Abdominal US	24.6%
		M: 44.2%				M: 35.1%
		F: 55.8%				F: 16.3%
Suzuki *et al*.^12^	Ishikawa prefecture	1537	35	1997–2002	Elevated transaminases	9.3%
		M: 73.2%				M: 10.5%
		F: 26.8%				F: 0.5%

F: female; M: Male; NR: Nonreported; NAFLD: Non‐alcoholic fatty liver disease; NASH: Non‐alcoholic steatohepatitis; US: Ultrasound.

We reported the only study reporting the prevalence of NASH in Japan.^8^ The authors used the validated scoring systems fibrosis index based on the 4 factors (FIB‐4) index (cut‐off ≥2.67) and body mass index, aspart aminotransferase, age, triglycerides (BAAT) index (cut‐off ≥3) to estimate the prevalence of NASH in the general population: 1.9 and 2.7%, respectively.^8^

Of the seven prevalence studies reported in Table 1, **s**ix reported prevalence stratified by gender, showing that, among the general Japanese population, the prevalence of NAFLD is consistently higher among males than females.^7^, ^8^, ^9^, ^11^, ^12^ In addition, except the studies conducted by Suzuki *et al*. and Hamaguchi *et al*., evidence shows that gender‐specific prevalence estimates are relatively consistent in Japan: 35.1–41.0% among males and 16.3–22.0% among females.^11^, ^12^

Although prevalence is higher among males, females are overrepresented in terms of advanced disease. Hashimoto *et al*. assessed 247 patients (male = 130) with NAFLD diagnosed by biopsy between 1990 and 2004.^13^ Females were found to comprise the majority of patients with advanced fibrosis or cirrhosis (stage F3/F4–56% female) but the minority of those with mild (F0/F2) disease (42%).^13^ A study conducted nationwide that indexed patients with biopsy‐proven cirrhosis reported that, among patients with nonviral liver cirrhosis, 24% of female patients, but only 9.5% of male patients, had NASH as the determining etiology as opposed to other cirrhosis etiologies.^14^ This result was confirmed in another nationwide study, which reported that more females with all‐cause cirrhosis had NASH etiology than males: 3.4 *versus* 1.4%, respectively.^15^

### *Incidence of* *NAFLD/NASH*

The incidence of NAFLD in healthy cohorts was reported in three studies, while one study reported the incidence of NASH among NAFLD patients.^11^, ^12^, ^16^ Suzuki *et al*. reported the incidence of elevated transaminases among a healthy general check‐up population as being 31 cases per 1000 person‐years of follow‐up.^12^ It is unclear whether this result reflects the true incidence of NAFLD in the Japanese population given the sole use of elevated transaminases as a proxy. Nevertheless, a study using abdominal United States (US) to estimate the incidence of NAFLD in the general health check‐up population reported a higher incidence rate.^11^ Hamaguchi *et al*. reported that, among a cohort of 3147 healthy middle‐aged participants, 308 developed NAFLD over a median follow‐up of 414 days, which estimates 86 cases per 1000 person‐years.^11^ Of 704 participants with NAFLD at baseline, 16% experienced disease resolution after a 1‐year follow‐up. Weight gain and presence of metabolic syndrome components (e.g. elevated BMI, blood pressure, fasting blood glucose, triglycerides, and decreased high‐density lipoprotein cholesterol [HDL‐C]) were predictive factors for the onset of NAFLD, while weight loss was associated with disease resolution.^11^

Tsunoda *et al*. reported the incidence of NASH among 1149 patients with NAFLD, as diagnosed with ultrasound and liver enzyme levels (alanine aminotransferase [ALT] and aspartate aminotransferase [AST]).^16^ During a mean follow‐up of 4.2 years (4804 person‐years in total), 318 participants (27.7%) progressed from NAFLD to NASH, which equals an incidence rate of 66.19 cases per 1000 person‐years.^16^

## Prevalence of comorbidities

### *Obesity*

Obesity was the most commonly prevalent comorbidity of NAFLD/NASH in Japan. In most studies, obesity was defined as BMI ≥25 kg/m^2^, and prevalence estimates ranged from 65 to 81% across broad NASH patients in all included studies.^17^, ^18^, ^19^, ^20^, ^21^, ^22^, ^23^, ^24^, ^25^

**Figure 1:**
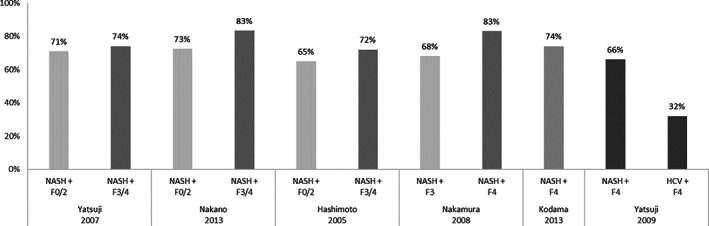
Prevalence of obesity among non‐alcoholic steatohepatitis (NASH) patients depending on fibrosis stage.

When stratifying by level of fibrosis, the prevalence of obesity among NASH patients ranged from 65 to 73% among patients with mild fibrosis and 72–83% among patients with advanced fibrosis (F3/F4 disease) as displayed in Fig. 1.^13^, ^17^, ^22^, ^24^, ^26^, ^27^ Studies conducted by Hashimoto *et al*. and Nakano *et al*. show a higher prevalence of obesity among patients with advanced disease (F3/F4) when compared to patients with mild fibrosis (F0–F2) (72 *vs* 65% and 83 *vs* 73%, respectively); however, these results failed to reach statistical significance.^13^, ^17^ Yatsuji *et al*. reported similar results; nevertheless, the authors only assessed statistical significance when stratifying results by age.^22^ According to study results, the prevalence of obesity was 80 and 67% in patients younger and older than 55 years old, respectively (*P* < 0.05), and the prevalence of morbid obesity (defined as BMI ≥30 kg/m^2^ in Japan) was reported as 36.6 *versus* 16.3% (*P* < 0.001) in younger and older patients, respectively.^22^ In NASH patients younger than 55 years old, obesity was found to be more prevalent in F0/2 patient than in F3/4 patients (82 *vs* 75%); nevertheless, statistical significance was not reached (*P* = 0.464).^22^

The prevalence of obesity in patients with NASH‐related cirrhosis ranged from 66 to 83% according to the results of three studies.^24^, ^26^, ^27^ Nakamura *et al*. reported a higher prevalence of obesity among patients with NASH‐related cirrhosis compared to NASH patients with advanced fibrosis (83 *vs* 68%); however, the authors did not assess statistical significance.^24^

Yatsuji *et al*. further found that obesity was present in 66% of patients with NASH‐related cirrhosis *versus* 32% of Hepatitis C virus (HCV)‐related cirrhosis patients (*P* < 0.001).^27^ This also suggests that obesity is characteristic of severe liver disease associated with NASH compared to other etiologies.

Among patients with NASH‐related HCC, obesity was prevalent in 62–84% of patients according to the results of four studies.^20^, ^28^, ^29^, ^30^

The characteristics of nonobese NAFLD/NASH patients were reported in three studies.^9^, ^25^, ^31^ In a study conducted among patients recruited from the general Japanese population through a health check‐up, Nishioji *et al*. recruited 3271 patients and estimated that the overall prevalence of NAFLD was 24.6%: 68.5% in obese subjects and 15.2% in nonobese subjects (lean‐NASH).^9^ The authors reported a mean BMI of 23 and 20.3 kg/m^2^ for male and female nonobese NAFLD patients, respectively. Honda *et al*. observed 540 NAFLD patients and compared the difference between obese (*n* = 406) and nonobese patients (*n* = 134).^31^ Steatosis grade, lobular inflammation, hepatocyte ballooning, and NAS were significantly lower in nonobese NAFLD compared with obese NAFLD patients, contrary to fibrosis stage, where a significant difference was not found.^31^ Similar results were reported by Ikarashi *et al*. when observing the clinical characteristics of 808 biopsy‐proven NASH patients and compared the clinical characteristics of the disease according to the following BMI ranges: BMI <22 kg/m^2^ (lean‐NASH); BMI >22 and <25 kg/m^2^ (normal weight NASH); and BMI ≥25 kg/m^2^ (obese NASH).^25^ The prevalence rate of obesity was reported to be 65% (524/808), with 33% of the nonobese patients classified as “lean‐NASH” (91/284). Comorbidities such as T2DM, dyslipidemia, hypertension, and steatosis grade 3 were more prevalent among patients with higher BMI.^25^ However, advanced fibrosis was found to be higher in lean‐NASH patients when compared to NASH patients with normal weight (45 *vs* 39%), despite lower lobular inflammation (58 *vs* 75%).^25^

### *Type 2 diabetes mellitus*

T2DM was also quite common among NASH patients, with prevalence rates ranging from 33 to 71%.^18^, ^20^, ^21^, ^22^, ^23^, ^24^, ^32^, ^33^, ^34^, ^35^, ^36^, ^37^ DM prevalence was higher in NASH patients compared to NAFLD patients according to the results of four studies:

- Seko *et al*. 2017 (*n* = 238): 54 *versus* 24% (*P* = 0.001)^33^
- Seko *et al*. 2015 (*n* = 312): 42 *versus* 18% (*P* = 0.001)^34^
- Yasui *et al*. 2011 (*n* = 174): 36 *versus* 24% (*P* = 0.140)^20^
- Tada *et al*. 2018 (*n* = 170): 52 *versus* 45% (*P* = 0.472)^37^

Statistical significance was found in studies that used larger sample sizes.

**Figure 2:**
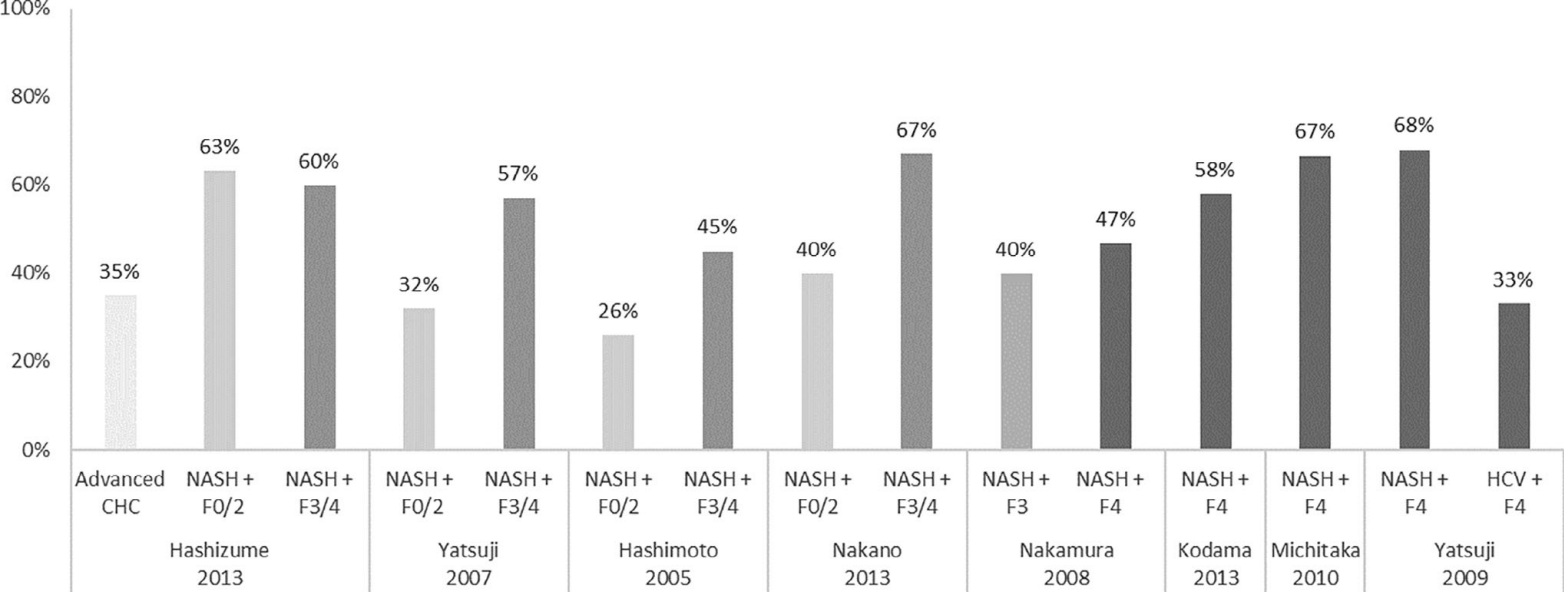
Prevalence of diabetes mellitus (DM) across non‐alcoholic steatohepatitis (NASH) patients depending on fibrosis stage.

Results from six studies suggest that DM is more prevalent in NASH patients with more advanced disease compared to those with milder disease, as displayed in Figure 2.^13^, ^15^, ^17^, ^22^, ^24^, ^26^, ^27^, ^38^

When comparing the prevalence of DM across fibrosis severity, four authors reported that DM was more prevalent in patients with worse fibrosis stage, while one author reported that DM was slightly more prevalent among patients with mild fibrosis compared to patients with advanced fibrosis (Hashizume *et al*., *P* = 0.899).^13^, ^17^, ^22^, ^24^, ^38^ Hashimoto *et al*. (45 *vs* 26%, *P* = 0.01) and Yatsuji *et al*., when assessing a subcohort of patients younger than 55 years old (50 *vs* 25%, *P* = 0.019), reported a statistically significant prevalence of DM in NASH patients with advanced fibrosis compared to mild fibrosis.^13^, ^22^ Among patients with NASH‐related cirrhosis, DM prevalence ranged from 47 to 68% according to the results of four studies.^15^, ^24^, ^26^, ^27^ Moreover, DM was significantly more prevalent in patients with NASH‐related cirrhosis compared to non‐NASH‐related cirrhosis according to Hashizume *et al*. and Yatsjui *et al*.^27^, ^38^ The prevalence of DM in patients with NASH‐related HCC was reported in six studies and ranged from 38 to 63%.^20^, ^30^, ^39^, ^40^, ^41^, ^42^

### *Dyslipidemia*

**Figure 3:**
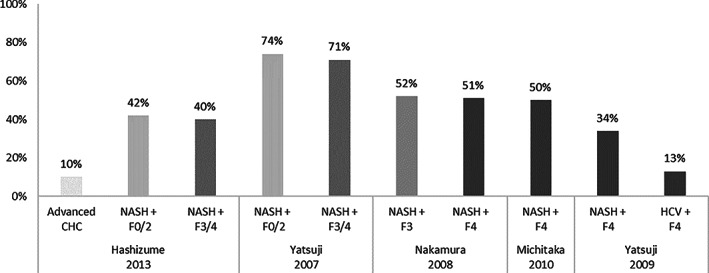
Prevalence of hyperlipidemia across non‐alcoholic steatohepatitis (NASH) patients depending on fibrosis stage.

Dyslipidemia and its most common form, hyperlipidemia, are also frequently found in NAFLD and NASH patients. Two studies compared the prevalence of hyperlipidemia in subjects with NAFLD *versus* controls without fatty liver, and in both studies, hyperlipidemia prevalence was significantly higher in male and female NAFLD patients compared with controls.^7^, ^43^ The prevalence of hyperlipidemia was also found to be higher among NASH patients compared to non‐NASH patients; nevertheless, this condition seemed to be independent of fibrosis progression as the prevalence rate of this condition was comparable between patients with mild fibrosis compared to patients with advanced fibrosis as displayed in Figure 3.^15^, ^22^, ^24^, ^27^, ^38^

### *Hypertension*

Across all NASH patients, the prevalence of hypertension ranged from 15 to 67%.^14^, ^18^, ^20^, ^22^, ^23^, ^24^, ^25^, ^32^, ^34^, ^35^, ^37^, ^44^, ^45^ According to two studies (Yasui *et al*. and Tada *et al*.), this condition was more frequent among NASH patients than NAFLD patients.^20^, ^37^ Hypertension was also more prevalent among obese patients as reported by Ikarashi *et al*. and Seki *et al*.: 54 and 67%, respectively.^25^, ^45^

**Figure 4:**
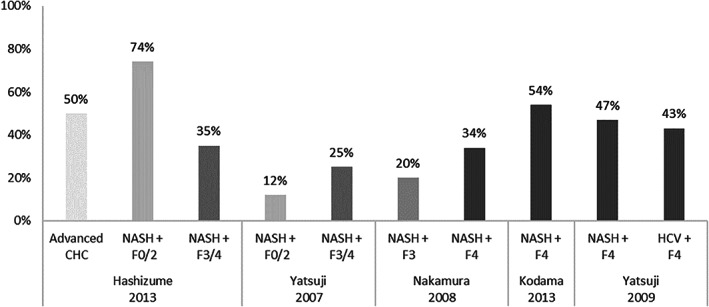
Prevalence of hypertension across non‐alcoholic steatohepatitis (NASH) patients depending on fibrosis stage.

Seven studies reported the prevalence of hypertension according to fibrosis stages (see Fig. 4 **).** 13, 17, 22, 24, 26, 27, 38 Across all studies, cohorts reporting higher prevalence rates of hypertension were generally older and reported higher mean BMI, as well as other comorbid conditions such as hyperlipidemia/dyslipidemia or chronic kidney disease (CKD).^13^, ^17^, ^22^, ^24^, ^26^, ^27^, ^38^

Hashizume *et al*. reported a significantly higher prevalence of hypertension in NASH patients with mild fibrosis compared with advanced fibrosis: 74 *versus* 35% (*P* = 0.025).^38^ Contradictory results were reported by Hashimoto *et al*.: 13 *versus* 29% (*P* = 0.01).^13^ The remaining studies comparing the prevalence of hypertension according to fibrosis stage did not report statistical significance, except in Nakamura *et al*., where it was not assessed.^17^, ^22^, ^24^, ^26^, ^27^

### *Metabolic syndrome*

Three studies reported the association of metabolic syndrome with NAFLD and NASH.^39^, ^46^, ^47^ Hamaguchi *et al*. evaluated a large prospective cohort of nominally healthy patients undergoing health check‐ups.^47^ Metabolic syndrome was present in 38% of patients with NAFLD (119/312) *versus* 6.5% in patients without NAFLD (87/1335).^47^ Furthermore, 40% of males and 26% of females with NAFLD had ≥3 components of metabolic syndrome at baseline compared to 8 and 3% of males and females without NAFLD, respectively. The presence of metabolic syndrome at baseline was significantly associated with NAFLD development during follow‐up (mean follow‐up: 414 days).^47^

Hashimoto *et al*. and Hashizume *et al*. evaluated the clinical characteristics of small cohorts of NASH patients with HCC and primary liver cancers, respectively.^39^, ^46^ In both cases, all patients except one (14/15 [93%] and 8/9 [89%]) met the criteria for metabolic syndrome, indicating an association between metabolic syndrome and progression from NASH to HCC/liver cancer.^39^, ^46^

### *Fibrosis staging*

Across eight studies reporting the fibrosis stage of NASH patients, mean fibrosis stage ranged from 1.30 to 2.09 using Brunt^19^, ^23^, ^33^, ^34^, ^35^, ^37^, ^45^, ^48^ and Younossi^19^, ^23^, ^33^, ^34^, ^35^, ^37^, ^45^, ^48^ scoring criteria. Two studies assessed the evolution of fibrosis in NASH patients treated with pharmacological therapy.^19^, ^48^ Hyogo *et al*. conducted an open‐label pilot study indexing 31 biopsy‐proven NASH patients with hyperlipidemia.^19^ All patients were treated with atorvastatin (10 mg daily) for 24 months. Follow‐up liver biopsy was performed in 17 patients, and although liver steatosis and NAFLD activity score (NAS) significantly improved, significant fibrosis stage improvement was not reported.^19^ Yoneda *et al*. indexed 10 biopsy‐proven NASH patients with dyslipidemia.^48^ The patients were given ezetimibe (10 mg/day) for 6 months, and after follow‐up biopsy, NAS and steatosis grade significantly improved.^48^ Fibrosis stage did not significantly change despite 6 of the 10 patients reporting an improvement in fibrosis stage.^48^

**Figure 5:**
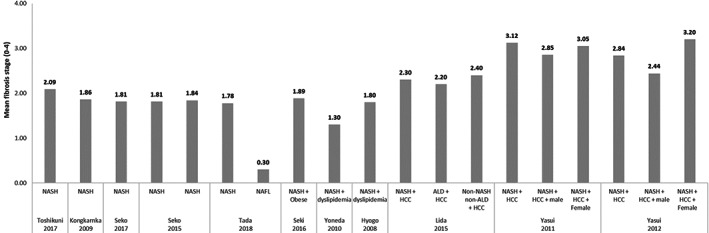
Mean fibrosis stage across broad non‐alcoholic steatohepatitis (NASH) patients *versus* NASH‐ hepatocellular carcinoma (HCC) patients.

In three studies reporting the mean fibrosis stage of patients with NASH‐related HCC, the score ranged from 2.30 to 3.20 based on Brunt^30^, ^40^, ^49^ and New Inuyama scoring.^30^, ^40^, ^49^ Iida *et al*. observed the fibrosis stage of HCC patients according to HCC etiology and reported no significant difference in mean fibrosis between NASH (2.30), alcoholic liver disease (ALD) (2.20), and non‐NASH non‐ALD (2.40).^40^ In two studies conducted by Yasui *et al*., mean fibrosis stage was found to be lower among male patients compared to female patients, with Yasui *et al*. reporting statistical difference between cohorts (*P* < 0.05).^30^, ^49^ The mean fibrosis stage of broad NASH patients and NASH‐HCC patients is summarized in Figure 5.

**Figure 6:**
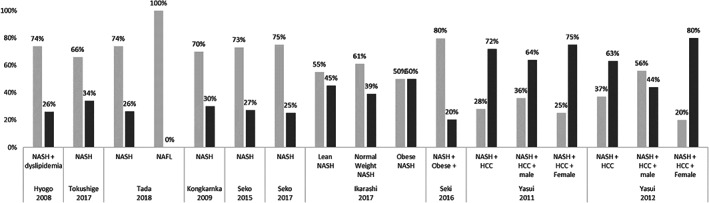
Distribution of mild and advanced fibrosis across non‐alcoholic steatohepatitis (NASH) and NASH‐related hepatocellular carcinoma (HCC) patients.

The presence of advanced fibrosis ranged from 21 to 50% among NASH patients and from 44 to 80% among patients with NASH‐related HCC.^19^, ^23^, ^25^, ^33^, ^34^, ^35^, ^37^ Seko *et al*. observed the distribution of fibrosis stages across 196 patients with biopsy‐proven NASH, diagnosed between 1999 and 2014.^34^ Of the initial cohort, 52 patients underwent a second biopsy (median time to the second biopsy was not provided by the authors), with 13 patients (25%) showing fibrosis progression of more than one stage, 26 patients (50%) showing no evolution, and 13 patients (25%) showing fibrosis regression.^34^ The overall annual rate of fibrosis stage change was 0.002/year. According to the authors, deterioration of NAS was correlated with progression of fibrosis stage (*P* < 0.05). Progression of fibrosis stage was observed in 8 of 16 (50%) patients with deterioration of NAS and in 5 of 36 (13.9%) patients without deterioration of NAS.^34^ The distribution of mild and advanced fibrosis across patients with NASH and NASH‐related HCC is displayed in Figure 6.

## Discussion

This systematic literature review shows that, despite some evidence gaps, the epidemiology of NAFLD and NASH in Japan is similar to Western countries. Since the early 1990s, the prevalence of NAFLD has increased at least twofold, with current estimates ranging from 24.6 to 34.7%, which is consistent with most Western countries.^70^ The only estimates that deviate from the presented ranges are the studies conducted by Hamaguchi *et al*. and the study of Suzuki *et al*.^11^, ^12^, ^47^ Suzuki *et al*.’s low estimate likely results from the different diagnosis method used by the authors (elevated aminotransferases as a proxy for NAFLD instead of abdominal US), as well as a notably younger cohort compared to the remaining studies.^12^ The reasons for the relatively low estimates from Hamaguchi *et al*. are not apparent as method of diagnosis, cohort age, and mean BMI are comparable to the other cohorts.^11^, ^47^ Both studies were undertaken in Gifu prefecture, and it is unclear whether this region of the country has lower prevalence of NAFLD compared to others. While estimates are consistent and are based on large population‐based studies, they rely on diagnosis by ultrasound and are unable to provide biopsy‐based estimates of NASH prevalence. A single study using validated scoring systems suggests that up to 2.7% of the adult general population may have NASH.^8^ This estimate is also consistent with non‐biopsy‐proven estimates of NASH in Europe and the United States.^70^ Nevertheless, the true prevalence of NASH in Japan is likely to be underestimated as studies conducted worldwide using liver biopsy as the method for NASH show that approximately 20–33% of NAFLD patients have NASH.^71^ Outside the research period of this study, two studies indicate that the numbers may be even higher. Miyake *et al*. performed a cross‐sectional study on Japanese subjects with low urine pH (<7.5). Within this subset, the authors estimated the prevalence of NAFLD to be 44.2% for men with urine pH ≤5.5 and 36.7% >5.5.^72^ Furthermore, in a single‐center study by Yamamura *et al*. in Japan on the profiles of advanced hepatic fibrosis, the authors state that 51.4% of their subjects suffered from fatty liver disease.^73^ These recent studies showing a steady increase of NAFLD in the Japanese population may indicate a further increase of NAFLD prevalence in the future.

In contrast to prevalence estimates, there is a paucity of evidence regarding the incidence of NAFLD and NASH. In the four studies assessing incidence, there was heterogeneity in the measures used to report incidence, cohort baseline characteristics, length of follow‐up, and method of diagnosis. Among subjects who underwent a general health check‐up, incidence rates were estimated at 31 cases per 1000 person‐years in a study that used elevated transaminases as the method of NASH diagnosis and at 86 cases per 1000 person‐years in a study that used elevated transaminases as the method of NASH diagnosis.^11^, ^12^

NAFLD and NASH are consistently associated with several metabolic comorbidities in the Japanese population. While the presence of NAFLD and NASH were reliably associated with obesity, T2DM, hypertension, hyperlipidemia, and metabolic syndrome, only obesity, T2DM, and metabolic syndrome appeared to be reliably associated with disease severity. Obesity was highly prevalent among NASH patients and more prevalent among those with advanced disease, suggesting a link with disease progression. NASH‐related cirrhosis patients had higher rates of obesity than cirrhosis due to other etiologies.^13^, ^17^, ^20^, ^27^, ^30^ However, 20–35% of patients in the assessed cohorts were not obese, suggesting that lean‐NASH plays an important role in Japanese NASH epidemiology.^9^, ^25^, ^31^ There was a paucity of studies assessing the clinical characteristics or prognosis of lean NASH, representing an evidence gap. Japanese NASH patients with worse fibrosis states also reported higher prevalence rates of DM, which might indicate that DM might be associated with disease severity.^15^, ^24^, ^26^, ^27^ Metabolic syndrome itself was only assessed in three studies. In all three studies, there was a higher prevalence of metabolic syndrome among NAFLD/NASH patients than in controls, and disease severity appeared to correlate with the number of metabolic syndrome components reported.^39^, ^46^, ^47^

The reported prevalence of hypertension was relatively high among Japanese NASH patients, with two studies reporting higher prevalence rates in NASH patients compared to NAFLD.^20^, ^37^ Nevertheless, the prevalence of hypertension was not consistent with disease severity. The reasons for these disparities were not apparent based on cohort characteristics or method of diagnosis, with hypertension appearing to be associated with other age and comorbid conditions (e.g. obesity and CKD) rather than disease severity per se.^13^, ^17^, ^22^, ^24^, ^26^, ^27^, ^38^ Although NAFLD/NASH patients reported higher prevalence rates of hyperlipidemia than non‐NAFLD/NASH patients, hyperlipidemia seemed to be independent of fibrosis progression as the prevalence rate of this condition was lower in more advanced fibrosis stages.^15^, ^22^, ^24^, ^27^, ^38^ Further large‐size prospective cohort studies are necessary to confirm the trends observed between comorbidities and disease progression.

Patients with NASH‐HCC reported higher weighted mean fibrosis stage than NASH patients, who in turn had more advanced fibrosis than NAFLD patients.^30^, ^49^ The use of cholesterol‐lowering agents may be associated with a decrease in fibrosis stage; however, results were based on studies with small sample sizes.^19^, ^48^ Further studies are required to determine whether use of anticholesterol agents is a viable strategy for disease resolution.

Overall, this review shows the high and increasing prevalence of NAFLD and NASH in the Japanese population. These NAFLD/NASH patients generally suffer from a high number of comorbid conditions, some of which are associated with disease progression. Thus, early diagnosis and treatment of NAFLD/NASH patients may be warranted in Japan.

## Declaration of conflict of interest

Received funding from Gilead Sciences, Inc.

## Supporting information


**Appendix** S1: Supporting Information.
