# Supplementary material for: Epidemiology of non‐alcoholic fatty liver disease and non‐alcoholic steatohepatitis in Japan: A focused literature review
Source: JGH Open. 2020 May 5;4(5):808–17. doi: 10.1002/jgh3.12349 (PMC7578337; doi:10.1002/jgh3.12349)
Supplement: Supplementary file 1 — Appendix S1: Supporting Information. [file JGH3-4-808-s001.pdf]

Appendix 1 PICOS framework and search strategy

**PICOS Component**	**Epidemiology and natural history of NAFLD/NASH in Japan**
Population	Patients with NAFLD/NASH in Japan (primary focus on patients with F3 or F4 disease), including patients with lean NASH.
Intervention	No specific restrictions
Comparators	No specific restrictions
Outcomes	• Incidence<br>• Number of cases<br>• Prevalence<br>• Incidence and prevalence of co-morbidities<br>• Patient demographics and clinical characteristics, including presence of comorbidities (e.g. diabetes, cardiovascular and renal disease)<br>• Distribution of fibrosis stage
Study type	Observational studies (including, but not limited to: epidemiologic surveillance data, case-control studies, cohort studies, follow-up studies, longitudinal studies, cross-sectional studies)
Country of Interest	Japan
Language	English and Japanese

Search strategy for English literature

Search terms for EMBASE (articles, articles-in-press, reviews) via www.embase.com

Date of the search: 04/07/2018

#	Query	# hits
1	'nonalcoholic steatohepatitis':ti,ab OR 'non#alcoholic steatohepatitis':ti,ab OR ‘nash’:ti,ab	15,414
2	'nonalcoholic fatty liver'/exp OR ('f3<br>coholic fatty liver disease' OR 'non#alcoholic fatty liver disease' OR NAFLD OR 'non-alcoholic fatty liver' OR 'non#alcoholic fatty liver' OR NAFL):ti,ab	33,640
3	#1 OR #2	37,283
4	japan:ti,ab,ff,ca OR japanese:ti,ab,ff,ca OR tokyo:ti,ab,ff,ca OR kyoto:ti,ab,ff,ca OR yokohama:ti,ab,ff,ca OR osaka:ti,ab,ff,ca OR fukuoka:ti,ab,ff,ca OR kobe:ti,ab,ff,ca OR sapporo:ti,ab,ff,ca OR nagoya:ti,ab,ff,ca OR saitama:ti,ab,ff,ca	1,846,071
5	#1 AND #4	1,734
6	#3 AND #4	2,977
7	#5 AND ('article'/it OR 'article in press'/it OR 'review'/it)	1,001
8	#6 AND ('article'/it OR 'article in press'/it OR 'review'/it)	1,784

Search terms for EMBASE (conference abstracts, papers and reviews) via www.embase.com

Date of the search: 13/07/2018

#	Query	# hits
1	'nonalcoholic steatohepatitis':ti,ab OR 'non#alcoholic steatohepatitis':ti,ab OR ‘nash’:ti,ab	15,465
2	'nonalcoholic fatty liver'/exp OR ('non-alcoholic fatty liver disease' OR 'non#alcoholic fatty liver disease' OR NAFLD OR 'non-alcoholic fatty liver' OR 'non#alcoholic fatty liver' OR NAFL):ti,ab	33,775
3	#1 OR #2	37,434
4	japan:ti,ab,ff,ca OR japanese:ti,ab,ff,ca OR tokyo:ti,ab,ff,ca OR kyoto:ti,ab,ff,ca OR yokohama:ti,ab,ff,ca OR osaka:ti,ab,ff,ca OR fukuoka:ti,ab,ff,ca OR kobe:ti,ab,ff,ca OR sapporo:ti,ab,ff,ca OR nagoya:ti,ab,ff,ca OR saitama:ti,ab,ff,ca	1,848,348
5	#3 AND #4	2,982
6	#5 AND ([conference abstract]/lim OR [conference paper]/lim OR [conference review]/lim)	1,044
7	#6 AND [2015-2018]/py	473
8	[animals]/lim NOT [humans]/lim	5,393,326

9	#7 NOT #8	334

Search terms for MEDLINE and MEDLINE-IN-PROCESS via www.pubmed.com

Date of the search: 04/07/2018

#	Query	# hits
1	“Non-alcoholic fatty liver disease”[MH] OR “non-alcoholic fatty liver disease”[TIAB] OR “non?alcoholic fatty liver disease”[TIAB] OR “NAFLD”[TIAB] OR “non-alcoholic fatty liver”[TIAB] OR “non?alcoholic fatty liver”[TIAB] OR “NAFL”[TIAB]	14,475
2	“Nonalcoholic steatohepatitis”[TIAB] OR “non?alcoholic steatohepatitis”[TIAB] OR “NASH”[TIAB]	8,778
3	#1 OR #2	18,416
4	japan[MH] OR japan[TIAB] OR japan[AD] OR japan[PL] OR japanese[TIAB] OR japanese[AD] OR tokyo[MH] OR Tokyo[TIAB] OR Tokyo[AD] OR kyoto[TIAB] OR kyoto[AD] OR yokohama[TIAB] OR yokohama[AD] OR osaka[TIAB] OR osaka[AD] OR fukuoka[TIAB] OR fukuoka[AD] OR kobe[TIAB] OR kobe[AD] OR sapporo[TIAB] OR sapporo[AD] OR nagoya[TIAB] OR nagoya[AD] OR saitama[TIAB] OR saitama[AD]	1,697,790
5	#2 AND #4	1,167
6	#3 AND #4	1,822

Search strategy for Japanese literature

<u>Search terms for Ichushi Web</u>

Date of the search: 04/07/2018

#	Query	# hits
1	NASH	594
2	NAFLD	366
3	非アルコール性脂肪性肝炎	352
4	非アルコール性脂肪性肝疾患	349
5	非アルコール性脂肪性肝	95
6	非アルコール性脂肪性肝障害	1
7	非アルコール性脂肪肝炎	352
8	非アルコール性脂肪肝	360
9	非アルコール性脂肪肝疾患	14
10	#1 OR #2 OR #3 OR #4 OR #5 OR #6 OR #7 OR #8 OR #9	636

Appendix 2　PRISMA flowchart

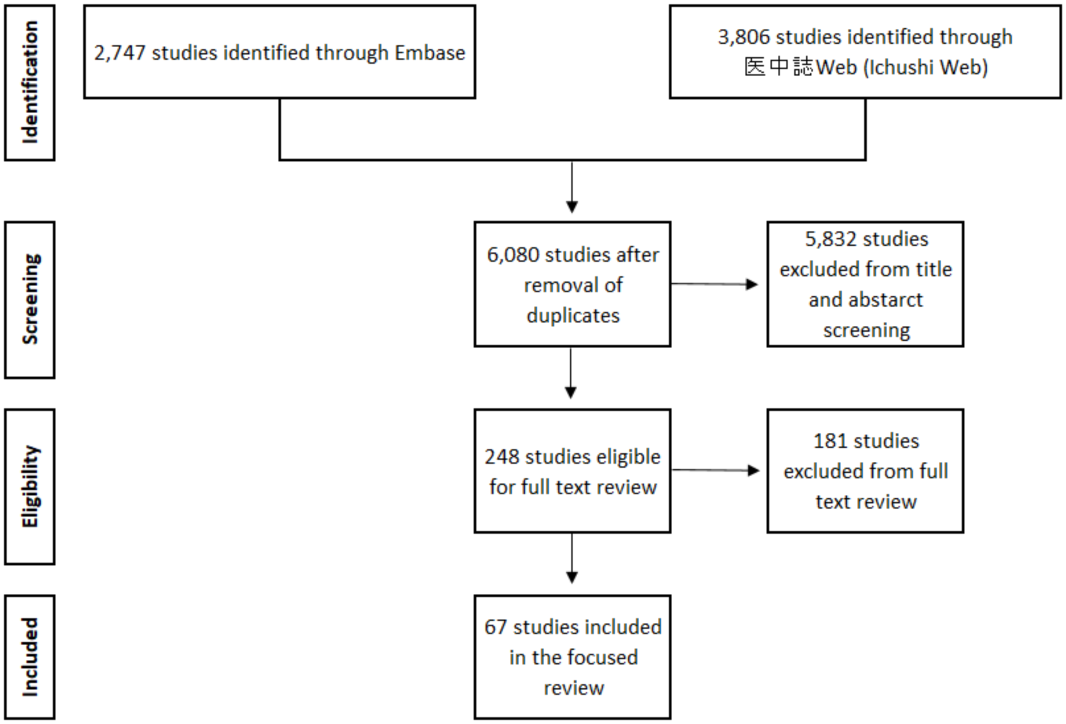

Appendix 3 Summary of study characteristics

Author	Type of study	Prefecture/ city	Data Source	Study Period	Population	Method of NAFLD/ NASH diagnosis	Sample Size	Mean/ median Age (years)	Gender (% males)
**Nishioji, 2015** [50]	Cross-sectional	Kyoto	Health care division of Kyoto Second Red Cross Hospital	2011 - 2012	General population	NR	3,271	NR	44%
**Jimba, 2005** [7]	Cross-sectional	Kurihashi	Saitama-ken Saiseikai Kurihashi (SSK) Hospital	2002 - 2003	Impaired glucose metabolism	Abdominal US	1,950	49	69%
**Hamaguchi, 2005** [11]	Prospective	Gifu	Gifu University School of Medicine	2002 - 2003	General population	Abdominal US	4401	47.6	59%
**Yatsuji, 2009** [27]	Prospective	Tokyo; Tokyo	Tokyo Women's Medical University	1990 - 2006	NASH + cirrhosis	Liver biopsy	68	62.7	43%
**Yasui, 2011** [20]	Cross-sectional	Kyoto and Nara	Hospital of Kyoto Prefectural University of Medicine and Nara City Hospital	2001 - 2006	NAFLD	Liver biopsy	174	54	59%
**Arase, 2011** [51]	Retrospective	Tokyo	Department of Hepatology and/or Health Management Center of Toranomon	1997 - 2007	NALFD	Liver biopsy	5,561	48	88%
**Arase, 2012** [52]	Retrospective	Tokyo	Department of Hepatology and/or Health Management Center, Toranomon	1997 - 2007	NALFD	Liver biopsy	3,200	62.5	75%
**Chan, 2018** [32]	Cross-sectional	Two centres in Japan	Yotsuya Medical Cube/Gunma University Graduate School of Medicine, and Yokohama City University School of Medicine	2009 - 2016	NAFLD and NASH	NAFLD – abdominal US NASH – Liver biopsy	112	NR	NR

**Eguchi, 2012** [8]	Retrospective	Three centres in Japan	Eguchi Hospital Health, Kawamura Clinic Heath Center, and Kochi Medical School Hospital	2009 - 2010	General population	Abdominal US	5,075	50	52%
**Hashimoto, 2004** [39]	Retrospective	Tokyo	Tokyo Women's Medical University	1989 - 2003	NASH + HCC	Liver biopsy	8	68	37%
**Hashimoto, 2005** [13]	Prospective	Tokyo	Tokyo Women's Medical University or an affiliated hospital	1990 - 2004	NAFLD	Liver biopsy	247	53	53%
**Hashimoto, 2009** [28]	Prospective	Tokyo	Tokyo Women's Medical University or an affiliated hospital	1990 - 2004	NASH + HCC	Liver biopsy	348	50	41%
**Hashizume, 2007** [46]	Retrospective	Maebashi	Gunma University Graduate School of Medicine and affiliated hospitals	2000 - 2005	NASH +HCC	NR	NR	NR	NR
**Hashizume, 2013** [38]	Prospective	Maebashi	Gunma University Graduate School of Medicine and affiliated hospitals	2003	NASH	NR	39	68	0
**Honda, 2016** [31]	Cross-sectional	National	University Hospitals of Hiroshima Kurume and Yokahoma	NR	NAFLD + non-obese vs obese	NR	134 vs 406	56.6 vs 48.5	43% vs 56%
**Hyogo, 2008** [19]	Prospective	Hiroshima	Hiroshima University Hospital	NR	NASH + dyslipidemia	NR	31	52.5	65%
**Iida, 2015** [40]	Retrospective	Meiwa	Meiwa Hospital	2001 - 2013	NASH + HCC	NR	40	71.6	73%
**Kang, 2018** [53]	Retrospective	Sapporo	A tertiary hospital in Sapporo	1998 - 2016	NAFLD + Cirrhosis	Abdominal US	100	NR	NR
**Kawada, 2009** [54]	Cross-sectional	Osaka	Osaka Medical Center for Cancer and Cardiovascular Diseases.	1990 - 2016	NASH + HCC	Abdominal US	807	73	NR
**Kawaguchi, 2018** [55]	Prospective	National	17 medical institutions in Japan	2000 - 2014	NAFLD +HCC	NR	247	71	65%
**Kinoshita, 2016** [42]	Prospective	Osaka	Osaka City University Hospital	1995 - 2014	NASH + mHCC	NR	34	58	56%

**Kikuchi, 2009** [56]	Retrospective	Meiwa	Meiwa Hospital	2001 - 2005	NASH + HCC	NR	10	70.91	70%
**Kawamura, 2012** [57]	Retrospective	Tokyo	Toranomon Hospital	1997 - 2010	NAFLD	Abdominal US	6,508	49	88%
**Tokushige, 2010** [29]	Retrospective	Tokyo	Tokyo Women's Medical University	1990 - 2007	NASH + HCC	Liver biopsy	34	70.14	62%
**Tokushige, 2015** [58]	Retrospective	National	15 tutorial hospitals in Japan	2000 - 2013	NAFLD + HCC	NR	209	72	70%
**Tokushige, 2013** [59]	Retrospective	National	National Survey	2006 - 2009	NAFLD + HCC	NR	292	72	62%
**Toshikuni, 2017** [35]	Retrospective	National	National Survey	2009 - 2011	NASH	NR	581	54	51%
**Wakai, 2011** [60]	Retrospective	Niigata	Niigata University Medical and Dental Hospital	1990 - 2007	NAFLD + HCC	NR	17	65	58%
**Yasui, 2011** [30]	Cross-sectional	National	15 hepatology centers in the Japan NASH Study Group	1993 - 2010	NASH + HCC	NR	87	72	62%
**Yasui 2012** [49]	Retrospective	Multicenter	NR	NR	NASH + HCC	NR	19	65	47%
**Yatsuji, 2009** [22]	Cross-sectional	Tokyo	Tokyo Women's Medical University	1991 - 2005	NASH	Liver biopsy	193	53	56%
**Kodama 2013** [26]	Prospective	Tokyo	Tokyo Medical University	1990 - 2010	NASH + Cirrhosis	Liver biopsy	72	63.5	32%
**Komeda 2005** [6]	Prospective	Kyoto	Kyoto University Hospital	N/R	NASH	Liver biopsy	26	56.6	38%
**Kongkarnka 2009** [23]	Retrospective	Nara	Nara Medical University	2003 - 2008	NASH	NR	50	54.66	52%
**Michitaka 2010** [15]	Retrospective	Nationwide	58 hospitals in Japan	N/R	NASH +cirrhosis	Liver biopsy	647	66.6	40%

**Nagaoki 2012** [41]	Retrospective	Hiroshima	Hiroshima University Hospital	1995 - 2009	NASH + HCC	NR	17	66.3	65%
**Nakajima 2015** [61]	Cross-sectional	N/R	N/R	N/R	NASH	NR	51	59	45%
**Kogiso 2014** [44]	Retrospective	Tokyo	Hiroshima University Hospital	1991 - 2012	NASH	NR	691	NR	NR
**Nakamura 2008** [24]	Prospective	Tokyo	Tokyo Medical University	1993 - 2006	NASH	Liver biopsy	72	62	49%
**Nakano 2013** [17]	Retrospective	Tochigi	Dokkyo Medical University	1991 - 2011	NAFLD	Liver biopsy	52	48	40%
**Osawa 2015** [62]	Retrospective	Okayama	Okayama University hospital	2011 - 2013	CAD/CABG	Computed tomography	414	64	51%
**Seki 2016** [45]	Prospective	Tokyo	Yotsuya Medical Cube	2009 - 2011	NAFLD + Obese + bariatric surgery	Liver biopsy	102	42.7	54%
**Seko 2015** [63]	Retrospective	Kyoto	Kyoto Prefectural University of Medicine	1999 - 2014	NASH	Liver biopsy	52	68	42.%
**Seko 2017** [33]	Retrospective	Kyoto	Kyoto Prefectural University of Medicine	1999 - 2013	NAFLD	Liver biopsy	312	59	51%
**Shima 2015** [64]	Retrospective	Osaka	Saiseikai Suita Hospital	2007 - 2012	NAFLD male vs female	Liver biopsy	570	51.6 vs 61.8%	NR
**Sumida 2013** [21]	Retrospective	Nara	Nara Medical University	2002 - 2008	NASH	Liver biopsy	17	67	24%
**Suzuki 2013** [73]	Retrospective	Yokohama	Yokohama City University Graduate School of Medicine,	2006 - 2007	NAFLD	Liver biopsy	36	56	36%
**Suzuki 2013** [14]	Retrospective	Nationwide	48 hospitals in Japan	2011	NASH	Liver biopsy	1,015	67	43%

**Tada 2018** [37]	Retrospective	Ogaki	Ogaki Municipal Hospital and Kochi Medical School	1999 - 2013	NAFLD	Liver biopsy	170	54.5	47%
**Yoneda 2010** [48]	Prospective	Yokohama	Yokohama City University Hospital	N/R	NASH + dyslipidemia	Liver biopsy	10	51	60%
**Ikarashi 2017** [25]	Cross-sectional	NR	NR	NR	Lean NASH vs Obese NASH	Liver biopsy	284 vs 524	57 vs 49	45% vs 58%
**Urabe 2015** [18]	Retrospective	Osaka	Osaka University Hospital	1998 - 2009	NASH	Liver biopsy	134	55.7	31%
**Hamaguchi 2007** [47]	Prospective	Gifu	Murakami Memorial Hospital,	1998	General population	Abdominal US	1,647	NR	NR
**Arase 2009** [65]	Retrospective	Tokyo	Toranomon Hospital,	1997 - 2007	NAFLD	Abdominal US	6,003	48.8	88%
**Nakamuta 2005** [66]	Prospective	Kyushu	Kyushu University Hospital	2003 - 2004	Steatosis LDLT donors	Liver biopsy	7	38.8	90%
**Oza 2009** [67]	Prospective	Eguchi	Eguchi Hospital	2007 – 2008	NAFLD	Abdominal US	61	62.6	49%
**Tsunoda 2016** [16]	Prospective	Tokyo	Meiji Yasuda Shinjuku Medical Center	2005 - 2007	NAFL	NR	4,840	NR	NR
**Nakagami 2009** [43]	Cross-sectional	Saitama	Saiseikai Kurihas	2002 - 2003	NAFLD + Male vs female	Abdominal US	402 vs 130	48 vs 51	NR
**Tada 2017** [68]	Retrospective	Ogaki	Ogaki Municipal Hospital	2006 - 2015	NAFLD	Abdominal US	4,073	61	54%
**Sumida 2011** [69]	Cross-sectional	Kyoto	Kyoto medical plant	2009 - 2010	NAFLD	Abdominal US	4,130	52.5	79%

**Suzuki 2005** [12]	Retrospective	Nationwide	NR	1997 - 2002	NAFLD	Blood tests	529	35	73%
**Hamaguchi 2005** [70]	Retrospective	Gifu	Murakami Memorial Hospital	2001 - 2003	NAFLD male vs female	Abdominal US	3,876	47.9 vs 51	63% vs 37%
**Nishioji 2014** [9]	Cross-sectional	Kyoto	Kyoto Second Red Cross Hospital	2011 - 2012	NAFLD	Abdominal US	3,271	59	67%
**Tateishi 2015** [71]	Retrospective	Nationwide	53 tertiary care centres in Japan	1991 - 2015	NAFLD	Abdominal US	5,326	70	75.5%
**Tokushige 2011** [72]	Cross-sectional	Nationwide	115 hospitals in Japan	2006 - 2009	NAFLD-HCC	Liver biopsy	14,530	72	62%

NR: Non-reported; NAFLD: Nonalcoholic fatty liver disease; NASH: Nonalcoholic steatohepatitis; US: Ultrasound; HCC: Hepatocellular carcinoma; mHCC: Metastatic Hepatocellular carcinoma
